# Stoichiometric Characteristics of Carbon, Nitrogen, and Phosphorus in Leaves of Differently Aged Lucerne (*Medicago sativa*) Stands

**DOI:** 10.3389/fpls.2015.01062

**Published:** 2015-12-09

**Authors:** Zhennan Wang, Jiaoyun Lu, Mei Yang, Huimin Yang, Qingping Zhang

**Affiliations:** State Key Laboratory of Grassland Agro-ecosystems, College of Pastoral Agriculture Science and Technology, Lanzhou UniversityLanzhou, China

**Keywords:** *Medicago sativa*, alfalfa, cut, ecological stoichiometry, stand age

## Abstract

Element concentration within a plant which is vital to function maintenance and adaptation to environment, may change with plant growth. However, how carbon (C), nitrogen (N), and phosphorus (P) vary stoichiometrically with stand growth, i.e., ages or cuts, was still untouched in perennial species. This study tested the hypothesis that lucerne (*Medicago sativa*) C:N, C:P, and N:P should change with stand age and cut. Leaf C:N, C:P, and N:P changed with stand age, showing various trends in different cuts of lucerne. Generally the greatest stoichiometric ratios were measured in 8 year stand and in the second cut. They were affected significantly and negatively by total N and P concentrations of leaf, but not by organic C concentration. There were significantly positive correlations among leaf C:N, C:P, and N:P. However, leaf C:N, C:P, and N:P were hardly affected by soil features. Conclusively, lucerne C, N, and P stoichiometry are age- and cut-specific, and regulated mainly by leaf N, P concentrations and stoichiometry. There are few correlations with soil fertility. To our knowledge, it is the first try to elucidate the stoichiometry in the viewpoint of age and cut with a perennial herbaceous legume.

## Introduction

Element concentration of a plant reflect the balance of carbon (C) fixation and nutrient uptake during plant growth, thus playing a fundamental role in function maintenance and adaptation to environment (Wright and Westoby, 2003; Imaran and Gurmani, 2011). The concentration generally changes with plant growth as the plant differs in photosynthetic capability and nutrition requirement at different growth stages. How the elements vary as the plant grows has attracted a lot of attentions.

In a plant, main elements change with plant growth in an element-specific way. Meharg and Killham (1990) reported that the C was mainly distributed into the root during the early stages of growth (before 4 weeks) in perennial ryegrass (*Lolium perenne*), while less was translocated into the root from 8 to 24 weeks. Thus more C remains in the shoot, resulting in higher C concentration as this grass grows older. Muchow et al. (1996) found stalk sucrose (representing C) concentration increased and nitrogen (N) concentration decreased both in the leaf and stalk (Wood et al., 1996) with the growth of sugarcane (*Saccharum officinarum*). The concentrations of leaf N and phosphorus (P) are negatively correlated with leaf lifespan of trees (Wright and Westoby, 2003). As lucerne (*Medicago sativa*) grows in one cut, the crude protein content generally decreases, indicating that the N concentration is reduced, while the contents of fibers and lignins (representing C) increase in the leaf and stem (Martiniello et al., 1997; Tyrolova and Výborna, 2008; Marković et al., 2012). The C fixation through photosynthesis leads to C accumulation and increases in concentration. Meanwhile, a rapid uptake of mineral nutrients at early stages leads to N and P accumulations and then a gradual dilution may result in the decreases in the concentrations as the plant grows (Marković et al., 2009; Yang and Luo, 2011). But the total C and N contents based on biomass increase with the stand age in a restored forest community (Hooker and Compton, 2003). The increases in total contents are the function of photosynthetic C fixation and nutrients uptake. These changes were generally observed in some annual species or in some perennial species within a growing season or year. However, for the perennial species, such changes in element concentrations are more complex due to longer lifespan and multiple times to use annually (i.e., to cut or to graze the forages). Thus to find out how element concentration changes with stand age and cut is of special importance to better understanding the adaptation of a perennial plant to environments.

Researches on change in element concentration with stand age or lifespan (Debell and Radwan, 1984; Wright and Westoby, 2003; Chen et al., 2004) were mostly conducted in trees. The foliar P concentration of *Pinus sylvestris* var. *mongolica* and red alder (*Alnus rubra*) appeared to decrease with age (Debell and Radwan, 1984; Chen et al., 2004), and for foliar N, it showed inconsistent change in *P. sylvestris* var. *mongolica* (Chen et al., 2004) or decreased in red alder (Debell and Radwan, 1984) with stand age. This may be because of the increased supply of soil nutrient with date, and the increased adaptability of these species with the increased age (Chen et al., 2004). For some perennial species which are generally used as forages (i.e., lucerne), the frequent removal of shoots with cutting or grazing may lead to the change in element concentration within the plant. So in such perennials, the changing profiles of elements with age may change due to artificial utilization. After cutting or grazing, organic and soluble C concentrations of stubble and root would decrease quickly and then increase and the N concentrations would decrease slowly and then increase (Johansson, 1993; Ourry et al., 1994; Yang et al., 2013). Frank (2008) indicated that animal grazing increased shoot N concentration but had no effect on P concentration. For cut or grazed forages, the regrowth depends mainly on the reserved carbohydrates and nitrogen-containing compounds in the residue at the early stages, while at the later stages, the newly assimilated carbohydrates and absorbed nitrogen play more vital roles (Yang et al., 2013). However, changes in the concentrations of sole elements gave no full description on how elements change with stand age and cut. To date, effects of stand age and cut on element concentration are far more than completely understood in the perennial species.

Considering all elements are closely related in the plant, measurement of multiple elements should be more efficient to express the change of element status with plant growth. Stoichiometric ratio is an excellent measure to study multiple elements at a time within the plant and the balance of multiple chemical substances in ecological interactions and process (Sterner and Elser, 2002), playing an important role in elucidating the responses of a plant to diverse changes and the adaptation to different environments. In the plant, C:N and C:P represent the capacity of photosynthetical C fixation under N or P accumulation, and N:P can be used as the indicator to study plant nutrient restrictions in adverse habitats (Zhang et al., 2004). The ratios are easily influenced by plant phenotype, habitat feature, and human disturbance (Güsewell, 2004; Reich and Oleksyn, 2004; Han et al., 2005; Kerkhoff et al., 2005; Fu et al., 2007; Karalić et al., 2007; Zheng and Shangguan, 2007; Frank, 2008; He et al., 2008; Sardans et al., 2011; Yang et al., 2011a). Increased soil nutrient (Han et al., 2005), annual precipitation and decreased annual temperature (Han et al., 2005; Sardans et al., 2011) would increase plant nutrient concentration, and the ratios would change, following the variation in plant nutrient concentration. Wang et al. (2013) showed preliminarily that lucerne C:N was positively correlated with organic C (OC) concentration, while negatively correlated with total N (TN) concentration. Güsewell (2004) indicated that shoot biomass N:P increased with N:P in supply. Some studies have focused on the possible effects of growth stage and stand age on stoichiometric characteristics in plants. Wood et al. (1996) found that in sugarcane biomass:N increased with crop age throughout a growing season, possibly implying the increase in C:N. Duquesnay et al. (2000) indicated that leaf N:P of beech (*Fagus sylvatica*) was higher in the samples of 1996–1997 than 1969–1971. Hooker and Compton (2003) found that the C:N of the forest plant increased with the increased ages and this was approved by Yang and Luo (2011), while Clinton et al. (2002) found that the C:N of the forest plant did not significantly vary with age. Wang et al. (2014) indicated that leaf N:P of lucerne generally decreased and then rose with stand age. However, these studies focused more on trees and only C:N or N:P was measured, while little effort has been put on herbaceous perennials with measurements of all C:N, C:P, and N:P. The investigation on the stoichiometry with herbaceous perennials will help to better understand the adaptive mechanisms of terrestrial plants to environments.

Lucerne, a typical perennial legume forage with an average lifetime exceeding 20 years (Bennett, 2012), has been cultivated almost all over the world with an area of 32 million ha (Bouton, 2001). Extensive researches on lucerne adaptation in diverse environments will be of great importance in lucerne hay and seed production. Evidences on the change and balance of elements during lucerne growth have given some clues for further studying on lucerne adaptation. Marković et al. (2009) indicated that the N concentration was gradually diluted and the P concentration increased with growth in part or whole of lucerne. In a greenhouse experiment, lucerne C and N concentrations varied at different growing stages, and C:N dropped in the first cut, rose in the second cut and rose then dropped in the third cut for the leaf, while for the stem and root, it tended to drop and then rose in the first cut, rose in the second cut and rose and then dropped in the third cut (Wang et al., 2013). Zhang et al. (2013) indicated that the whole plant of lucerne N:P basically decreased and then rose with stand age. There were inconsistent results describing C:N and N:P changes with lucerne age and cut. The concentrations of N and P increased in the green leaves of lucerne, and decreased in the senesced leaves with stand age, and N:P decreased and then rose with stand age (Wang et al., 2014). These studies have revealed preliminarily that in lucerne, main elements, i.e., C, N, and P, may change with stand age or cut, but some more complete description on elemental changes still needs to be furthered.

In this study, we tested a hypothesis that stoichiometric ratios of C, N, and P should change with stand age and cut of lucerne. The specific objectives were to find out: (1) How the stoichiometric ratios of C, N and P differ among stand ages and cuts of lucerne; and (2) What are the relationships of stoichiometric ratios in lucerne with concentrations and ratios in lucerne and soil.

## Materials and Methods

### Description of the Study Site

The study was conducted at the Qingyang experimental station (35°40′N, 107°51′E, 1298 m a.s.l.) of Lanzhou University, which locates on the Loess Plateau of northwestern China and appears a typical continental climate. Mean annual precipitation is 561 mm and 70% of this total usually falls in July, August and September, making this area a typical rainfed region. Mean annual temperature is 8–10°C and total annual solar duration is 2300–2700 h. The soil is a Heilu soil (Entisol of FAO classification) which is a sandy-loam with 70% silt and 23% clay, representing the major cropping soil of this area.

A landrace of lucerne (*Medicago sativa* L. cv. Longdong) is grown in this station, which is widely sown in this area and used as forage production. Before establishment in the station, these lucerne fields were cultivated with cereal crops like maize (*Zea mays*) or winter wheat (*Triticum aestivum*). The stands are generally rainfed without irrigation and no fertilizers were applied to the fields. General cutting was applied three times per year (twice in the first year), as is typical for growers in the region (Shen et al., 2009).

Note this experimental station is a large land of 15 ha or so. Lucerne stands of different ages distributed separately in the station and for each age there were at least three fields as replicates. When sampling, we chose blocks of at least 3 m × 5 m in each replicate. It was not a typical randomized block design, but the large land area and replicate number were enough to minimize the effect of location difference. Additionally, we could not able to get enough stands of different ages exactly in the testing duration, for instance we couldn’t have an 11 or 8 year stands. So this experimental design and arrangement should promise the validity of all the tests.

### Plant and Soil Samplings

Lucerne stands were sampled at the early flowering stages in June 3 (the first cut), August 10 (the second cut) and October 23 (the third cut), 2012. In this year, the ages of the stands were 4 (established in 2009), 5 (established in 2008), 8 (established in 2005), and 11 year stands (established in 2002). For each aged stand, samples were taken from three different fields in the station. In each of the three replicates, at least 15 plants were chosen for the stand of one age. The samples (and soil samples as well) were brought back to the lab which is about 200 m away from the fields. Sampled leaves were oven-dried at 105°C for 10 min and then at 80°C for at least 48 h. Dried samples were ground into uniformly fine powder to pass through a 1.0-mm sieve.

Soil samples were taken separately from the depths of 0–10, 10–20, 20–30, 30–60, and 60–90 cm when plant samples were taken. The samples were then brought back to lab in less than 5 min and dried at 36°C (Wang et al., 2014). The dried samples passed through 2-mm and 0.25-mm sieves for measurements of available and total nutrients. Note some of ammonium nitrogen in soils might be released due to drying. But 36°C used in this test is not a very high temperature and should have not too much impact on soil ammonia, as in some pioneering studies 55°C (Knops and Tilman, 2000) or 60°C (Wuest and Gollany, 2012) used for soil drying has proved little effect on ammonia. In addition, the effect of drying worked evenly on all soil samples from different ages. So this whole protocol for soil drying would help minimize the negative effect on total soil nitrogen.

### Measurements and Calculations

The OC in the plant and soil was measured by potassium dichromate/sulphuric acid mixture titration method (Yeomans and Bremner, 1988). The TN in the plant and soil was measured by using the Semimicro–Kjeldahl method (Bremner and Mulvaney, 1982) with a Kjeldahl Auto-analyzer (KDN-102C, Shanghai, China). Total P (TP) in the plant and soil was determined colorimetrically (Du et al., 2011) with a spectrophotometer (UV-2102 PCS, Shanghai, China). Soil ammonium N(NH_4_^+^) and nitrate N (NO_3_^-^) were extracted in 2 mol L^-1^ KCl and then the concentrations were determined with a flow injection analyzer (FIAstar 5000, FOSS, Denmark). Soil available P (AP) was extracted in 0.5 mol L^-1^ NaHCO_3_ and the concentration was determined using the Olsen method.

The stoichiometric ratios of C, N, and P both in lucerne and the soil were calculated as OC vs. TN (C:N), OC vs. TP (C:P), and TN vs. TP (N:P).

### Statistical Analysis

The differences in the concentrations or stoichiometric ratios in stand ages, cuts of lucerne were analyzed using one-way repeated measures ANOVAs. Where there were no significant effects, the average was compared using a one-way repeated measures ANOVA. The linear correlations of C:N, C:P, and N:P with element concentration and stoichiometric ratio were analyzed with the model y = *a*x + *b*.

## Results

### Leaf C:N, C:P, and N:P in Differently Aged Lucerne Stands

**FIGURE 1:**
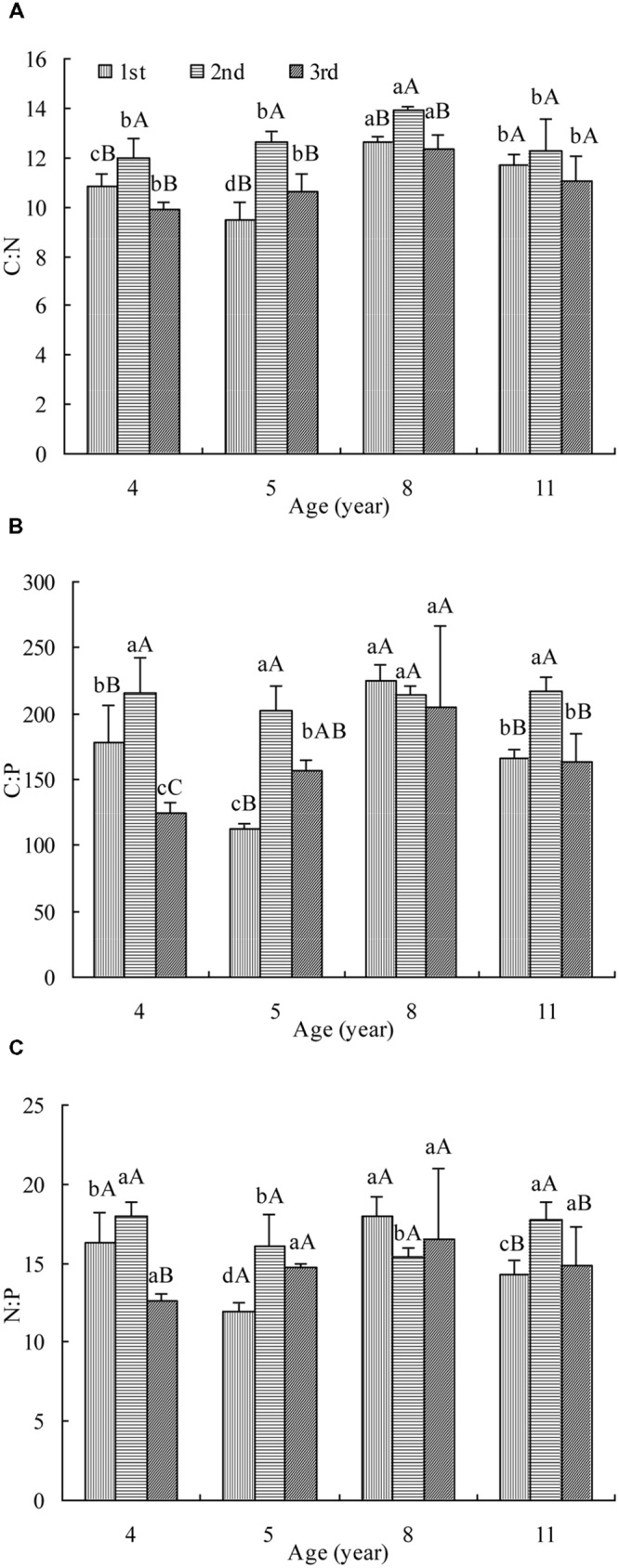
**The C:N **(A)**, C:P **(B)**, and N:P **(C)** in leaves of differently aged lucerne stands.** Different lowercase letters represent significant differences among ages at *P* < 0.05. Different capital letters represent significant differences among cuts at *P* < 0.05. The columns represent the arithmetic averages of the ratios, and the bars represent the standard deviations.

Leaf C:N was affected significantly by stand age and cut (**Figure 1A**). In the first cut, the C:N decreased firstly, then increased and finally dropped with age while in the rest two cuts, it increased and then decreased. The maximum C:N appeared in 8 year stand. The highest C:N was measured in the second cut of all ages and no significant difference was observed between the other two cuts. The tendency of C:N change with age and cut was generally the same as OC concentration (Supplementary Figure S1a) but opposite to TN concentration (Supplementary Figure S1b).

Leaf C:P was affected significantly by stand age and cut (**Figure 1B**). In the first cut, the C:P decreased firstly, then increased and finally dropped with age while in the third cut, it increased and then decreased. The maximum C:P of these two cuts was measured in 8 year stand. In the second cut, it hardly changed with age. The highest C:P was measured generally in the second cut of all but age 8. The tendency of C:P change with stand age and cut was opposite to TP concentration (Supplementary Figure S1c).

Leaf N:P was affected significantly by stand age and cut (**Figure 1C**). In the first cut, the N:P decreased firstly, then increased and finally dropped with age. In the second cut, it decreased and then increased with age while in the third cut, it hardly changed. It was generally the highest in the second cut of all but age 8. The tendency of N:P change was mostly opposite to that of TN or TP concentration in all but age 8 (Supplementary Figures S1b,c).

### Correlations of C:N, C:P, and N:P with OC, TN, and TP Concentrations in Leaves of Differently Aged Stands

Considering the average of both ages and cuts together, no correlation was observed between leaf C:N and OC concentration while negative correlations were observed between C:N and TN and TP concentrations (**Table 1**). Leaf C:N of averaged cuts was positively correlated with OC concentration in all and negatively correlated with TN concentrations while with TP concentration, it was only observed in 4 and 5 year stands. For C:N of averaged ages, no correlation was observed with OC concentration while negative correlations were observed with TN and TP concentrations (except TP of the second cut).

**Table 1:** Correlations of C:N, C:P, and N:P with OC, TN, and TP concentrations in leaves of differently aged lucerne stands.

	Cut	C:N					C:P					N:P				
		4	5	8	11	Average (age)	4	5	8	11	Average (age)	4	5	8	11	Average (age)
OC	Average (cut)	0.891^∗∗^	0.711^∗^	0.713^∗^	0.809^∗∗^		0.866^∗∗^					0.816^∗∗^				
TN	First					– 0.971^∗∗^					– 0.892^∗∗^					– 0.740^∗∗^
	Second					– 0.888^∗∗^										0.709^∗^
	Third					– 0.890^∗∗^					– 0.709^∗^					
	Average (cut)	– 0.850^∗∗^	– 0.968^∗∗^	– 0.691^∗^	– 0.865^∗∗^	– 0.912^∗∗^	– 0.852^∗∗^	– 0.898^∗∗^			– 0.755^∗∗^	– 0.838^∗∗^	– 0.704^∗^			– 0.481^∗∗^
	
TP	First					– 0.893^∗∗^					– 0.983^∗∗^					– 0.936^∗∗^
	Second										– 0.813^∗∗^					
	Third					– 0.680^∗^					– 0.961^∗∗^					– 0.935^∗∗^
	Average (cut)	– 0.925^∗∗^	– 0.845^∗∗^			– 0.773^∗∗^	– 0.967^∗∗^	– 0.969^∗∗^	– 0.980^∗∗^	– 0.945^∗∗^	– 0.964^∗∗^	– 0.986^∗∗^	– 0.931^∗∗^	– 0.926^∗∗^	– 0.947^∗∗^	– 0.889^∗∗^

*The data of all cuts are used for analyzing the correlations in a row and the data of all ages are used for analyzing the correlations in a column. Only the cases with significance are shown in the table. 4, 5, 8, and 11 refer to stand age in year and first, second, and third refer to the cut of one age. The asterisk (∗) shows significant correlations at *P* < 0.05 and double asterisks (∗∗) show significant correlations at *P* < 0.01.*

Negative correlations of leaf C:P of averaged both ages and cuts were observed with TN and TP concentrations while no correlation was observed with OC concentration (**Table 1**). The C:P of averaged cuts was positively correlated with OC concentration only in 4 year stand while negative correlations were observed with TN and TP concentrations (except TN of 8 and 11 year stands). For C:P of averaged ages, no correlation was observed with OC concentration while negative correlations were observed with TN and TP concentrations (except TN of the second cut).

Negative correlations of leaf N:P of averaged both ages and cuts were observed with TN and TP concentrations while no correlation was observed with OC concentration (**Table 1**). The N:P of averaged cuts was positively correlated with OC concentration only in 4 year stand while with TP concentration, negative correlations were observed in all ages. Dramatically, negative correlations were also observed with TN concentrations in 4 and 5 year stands. For N:P of averaged ages, no correlation was observed with OC concentration while with TP concentration, negative correlations were observed in the first and third cuts. Inconsistent correlations of N:P of averaged ages were also observed with TN concentration in the first two cuts.

### Correlations among C:N, C:P, and N:P in Leaves of Differently Aged Stands

Considering the average of both ages and cuts together, positive correlations were observed among C:N, C:P, and N:P in leaves (**Table 2**). Positive correlations were observed between N:P of averaged cuts and C:P, and positive correlation was observed with C:N only in 4 year stand. Positive correlations of C:P of averaged cuts were also observed with C:N in 4 and 5 year stands. For N:P of averaged ages, positive correlations were observed with C:P in the first and third cuts. There were inconsistent correlations between N:P of averaged ages and C:N in the first two cuts. Positive correlations were also observed in the first and third cuts between C:P of averaged ages and C:N.

**Table 2:** Correlations among C:N, C:P, and N:P in leaves of differently aged lucerne stands.

	Cut	C:P			N:P				
		4	5	Average (age)	4	5	8	11	Average (age)
C:N	First			0.881^∗∗^					0.714^∗∗^
	Second								–0.660^∗^
	Third			0.741^∗∗^					
	Average (cut)	0.983^∗∗^	0.892^∗∗^	0.799^∗∗^	0.941^∗∗^				0.476^∗∗^
	
C:P	First			1					0.958^∗∗^
	Third			1					0.940^∗∗^
	Average (cut)	1	1	1	0.986^∗∗^	0.924^∗∗^	0.928^∗∗^	0.844^∗∗^	0.907^∗∗^

*The data of all cuts are used for analyzing the correlations in a row and the data of all ages are used for analyzing the correlations in a column. Only the cases with significance are shown in the table. 4, 5, 8, and 11 refer to stand age in year and first, second, and third refer to the cut of one age. The asterisk (^∗^) shows significant correlations at *P* < 0.05 and double asterisks (^∗∗^) show significant correlations at *P* < 0.01.*

### Correlations of Leaf C:N, C:P, and N:P with Soil OC, NO_3_^-^ Concentrations and C:P

Positive correlation of leaf C:N was observed with soil OC concentration only in 30–60 cm (**Table 3**). And there was a positive correlation of leaf C:N of averaged ages in the third cut. Leaf C:N was positively correlated with soil NO_3_^-^ concentration only in 30–60 cm (**Table 3**). Positive correlations of leaf C:N of averaged cuts were observed with soil NO_3_^-^ concentration in 5 and 8 year stands. There was a positive correlation of leaf C:N of averaged ages with soil NO_3_^-^ concentration in the first cut. In addition, a positive correlation of leaf C:N and soil C:P was observed only in 30–60 cm (**Table 3**). And there was a positive correlation of averaged cuts with soil C:P in 4 year stand. In other cases, there was no significant correlation.

**Table 3:** Correlations of leaf C:N, C:P, and N:P with soil OC, NO_3_^-^ concentrations, and C:P.

	Soil depth (cm)	Cut	Leaf C:N				Leaf C:P				Leaf N:P			
			4	5	8	Average (age)	4	5	11	Average (age)	4	5	11	Average (age)
Soil OC	30–60	Third				0.647^∗^								
		Average (cut)				0.396^∗^				0.424^∗∗^				0.339^∗^
Soil NO_3_^-^	30–60	First				0.647^∗^								
		Average (cut)		0.765^∗^	0.796^∗^	0.549^∗∗^			0.856^∗∗^	0.435^∗∗^				
Soil C:P	30–60	Average (cut)	0.869^∗∗^			0.329^∗^	0.884^∗∗^	– 0.715^∗^	0.689^∗^	0.406^∗^	0.858^∗∗^	– 0.792^∗^	0.679^∗^	0.355^∗^

*The data of all cuts are used for analyzing the correlations in a row and the data of all ages are used for analyzing the correlations in a column. Only the cases with significance are shown in the table. 4, 5, 8, and 11 refer to stand age in year and first and third refers to the first and third cuts of one age. The asterisk (^∗^) shows significant correlations at *P* < 0.05 and double asterisks (^∗∗^) show significant correlations at *P* < 0.01.*

Leaf C:P was only positively correlated with soil OC concentration in 30–60 cm (**Table 3**). It was also positively correlated with soil NO_3_^-^ concentration in 30–60 cm (**Table 3**). Positive correlations was only observed between leaf C:P of averaged cuts with soil NO_3_^-^ concentration under 11 year stand. Additionally, leaf C:P was positively correlated with soil C:P in 30–60 cm (**Table 3**). Positive correlations were observed when it was with the average of cuts under 4 and 11 year stands, while negative correlation was observed in 5 year stand. In other cases, there was no significant correlation.

Leaf N:P was only positively correlated with soil OC concentration in 30–60 cm (**Table 3**). And leaf N:P was positively correlated with soil C:P in 30–60 cm (**Table 3**). Positive correlations were observed when it was with the average of cuts under 4 and 11 year stands, while negative correlation was observed in 5 year stand. In other cases, there was no significant correlation.

## Discussion

### Characteristics of C:N in Differently Aged Lucerne Stands

In lucerne, leaf C:N was affected significantly by stand age and cut. However, there was no monodirectional tendency of C:N change with stand age, differing from what reported in the forest plants (Hooker and Compton, 2003; Yang and Luo, 2011). They found that tissue C:N ratio of the forest plant increased significantly with stand age. This is because the increasing proportion of woody biomass leads to an increase in C:N ratio in plant tissue. However in this study, leaf C:N peaked at age 8 and then dropped. Lucerne is a perennial forage, so is a different species from trees. Importantly, the way lucerne stand is utilized may partly explain why it dropped after age 8. The frequent removal of shoots results in no “extra” C accumulation in the above ground part, differing from that in a tree the C accumulates as the stand grows more mature. So the leaf is always “newly emerged” after regrowth and OC concentration remains almost unchanged among stands of different ages (Supplementary Figure S1a). Obvious reduction in TN concentration was observed in age 8 and then TN concentration rose again in this study (Supplementary Figure S1b), explaining why C:N dropped after age 8. As a legume, lucerne has strong but different biological N fixation (BNF) in different stands with different ages which may lead to different N status in the leaf (Yang et al., 2011a), but BNF is easily affected by age. So it is assumed that the reduction of TN concentration may be attributed to weakened function of BNF. After age 8, different N nutrition status in the soil under lucerne (Supplementary Tables S1 and S2) might be one of the causes for TN concentration increase (Hooker and Compton, 2003) as lucerne has to use more soil mineral N if BNF is restricted. Combining with the lower C and N concentrations, the C:N peaked at age 8 suggested that not only the normal growth of lucerne was retarded but the N supply either from soils or BNF was weakened more seriously than C assimilation. In addition, leaf C:N was generally the greatest in the second cut of one age. The greater precipitation and higher temperature during the second cut may lead to more C accumulation through enhanced growth and photosynthesis (Zheng and Shangguan, 2007) and to diluted N concentration by rapid growth (Han et al., 2005). This “cut effect” on C:N may indicate that in practice, some optimal fertilization with N is necessary after cutting during the warmer season in this rainfed region.

The growing stages of lucerne among stand ages and cuts (in this study) and within each cut (Wang et al., 2013), which appear generally with different C and N status and with different functions to fix C and to assimilate N, may lead to different C:N and changing tendencies. In this study, there was no correlation between leaf C:N and OC concentration when considering the average of all ages and cuts, while only considering the average of three cuts of each age, there were positive correlations, indicating it is the age but not cut that affects the C:N more heavily. While negative correlations were observed with TN concentration in all cases, suggesting there is a strong solid connection between two values. Dramatically, there was a negative correlation between leaf C:N and TP concentration when considering the average of all ages and cuts, and some correlations considering the averages of ages or cuts. How TP change influences C:N is complex, but effect of P status on N status is obvious as a significantly positive correlation was observed between TN and TP concentrations in lucerne leaf (Supplementary Figure S2c). In another research, Hong et al. (2014) also indicated that leaf N and P concentrations were positively correlated.

However as a whole, the C:N indicates N use efficiency and represents the homeostatic regulation of C fixation and N assimilation (Sterner and Elser, 2002). So its change should not be interpreted simply with changes in OC and TN concentrations (Supplementary Figures S1a,b) and their interplays. In this study, there was no correlation between leaf OC and TN concentrations (Supplementary Figure S2a) while significant correlations of leaf C:N were observed with C:P and N:P, indicating their connections as intrinsic features of a plant.

### Characteristics of C:P in Differently Aged Lucerne Stands

In the forest, the increased woody biomass and the increased lignification maybe lead to the increase in tissue C:P significantly with stand age (Hooker and Compton, 2003; Yang and Luo, 2011). In lucerne, TP concentration is very much flexible among ages and generally the lowest at age 8 in this study (Supplementary Figure S1c). Considering that the OC concentration is stable (Supplementary Figure S1a), it is understandable that the C:P peaked at age 8 and then dropped. Thus, it suggested that at this age the growth was restricted and P uptake from soils into plants was reduced more, possibly due to shortage of N availability (as indicated by their positive correlation) and consequently weakness of P absorption. In addition, it was generally the highest in the second cut. This may be also attributed to the rapid growth due to the greater precipitation and higher temperature during this cut. Rapid growth of lucerne led to more C accumulation and diluted P concentration in the leaf.

The C:P indicates P use efficiency and represents a balanced C fixation and P assimilation in a plant (Sterner and Elser, 2002). So it is at least the consequence of changes in both OC and TP concentrations (Supplementary Figures S1a,c) and their interplays (Supplementary Figure S2b). In this study, change in lucerne leaf C:P was opposite to TP concentration, while the C:P hardly changed with leaf OC concentration. So, the TP concentration may affect C:P more heavily than OC concentration. Intriguingly, leaf C:P was negatively correlated with TN concentration, possibly resulting from the close link between N and P (Supplementary Figure S2c). Additionally, the C:P positively changed with C:N and N:P, indicating their roles in a plant as intrinsic features.

### Characteristics of N:P in Differently Aged Lucerne Stands

The leaf N:P was affected significantly by stand age and cut in this study. In the third cut, the N:P increased and then dropped with stand age from 4 to 11 years, which was the same as what was reported by Wang et al. (2014) but different from Zhang et al. (2013) where whole plant tissue was used. While in other cuts, it was very different. Both TN and TP concentrations changed a lot with stand age (Supplementary Figures S1b,c). That is partly because N and P are very active in a plant and easily influenced by stand age (Wang et al., 2014) or abiotic factors (Reich and Oleksyn, 2004; Han et al., 2005; Sardans et al., 2011). So the N:P was very flexible and the changing tendency with stand age was different among cuts. In addition, the N:P was generally highest in the second cut, which was opposite to TN or TP concentration (Supplementary Figure S1b,c). It suggested that the dilution to N or P is different and there may be more on P than N. The effect of high temperature on leaf TN and TP concentrations, and on N:P has been approved by Reich and Oleksyn (2004) in a global data with 5087 observations. Güsewell (2004) found that in *Molinia caerulea* and *Carex flava*, the shoot N:P was positively correlated with shoot N concentration and negatively correlated with shoot P concentration. Hong et al. (2014) also found the same correlations between N:P and N or P concentration in the roots and leaves across all species on the northern Tibetan Plateau. However in this study, leaf N:P was negatively correlated with both leaf TN and TP concentrations. It was not clear whether BNF plays a role in balancing N:P in leaves of lucerne (Yang et al., 2011a) which would have affected the correlation between N:P and TN concentration.

The N:P has proved a new, efficient but relatively easy way to assess plant N or P limitation (Aerts et al., 1992; Koerselman and Meuleman, 1996; Olde-Venterink et al., 2003; Güsewell, 2004; Zhang et al., 2004). However with different species living in different habitats, there were different thresholds of N:P for judging N or P limitation to growth. Considering there is some similar soil P background of this study in Qingyang as that of Zhang et al. (2004) in Inner Mongolia, the stands in Qingyang areas might be N-limited, in accordance with other reports of this group (Zhang et al., 2013; Wang et al., 2014). So in this area, optimal N fertilizer application is practical and will be helpful to sustain the persistent lucerne producton. However we still cannot tell whether BNF affects this judgment or not. Generally BNF should promise relatively enough N for lucerne growth so is impossible N-limited, but N loss due to frequent removal of shoots may lead to N deficiency during the regrowth. Their connection is under consideration and investigated in a new ongoing project.

### Soil-sourced Effectors and Effects on Stoichiometric Ratios of Lucerne C, N, and P

Plant N and P are derived from soil pool (or possibly BNF for legumes), so changes of N and P in soil pool should potentially affect plant C:N, C:P, and N:P. Considering these soil-sourced factors changed variously (Supplementary Tables S1, S2, and S3), their effect on leaf C:N, C:P, and N:P is complex. Cui et al. (2010) indicated that increasing N concentration of soil increased leaf N concentration. This would decrease plant C:N and increase N:P. Yang et al. (2011b) found that increased N deposition increased N concentrations in plant tissues and as a consequence, decreased C:N. Although there were some positive correlations between plant nutrient concentration and soil nutritional status, yet it did not mean there was a solid relationship between soil nutritional status and stoichiometric ratios in plants. In this study, few correlations of leaf C:N, C:P, and N:P were observed with soil OC, TN, TP, or available nutrients concentrations. Firstly, the ratios are closely and directly related to leaf nutrient concentration and soil fertility may affect leaf nutritional status as priority, so at least effect of soil fertility on the ratios is indirect and weak. Secondly, change in soil fertility generally affects multiple nutrients within the plant at a time (Sterner and Elser, 2002), thus leading to a stable ratios of these nutrients as they may change simultaneously. However, if some of the nutrients were scarce in the soil, there would be more interactions between leaf C, N, and P stoichiometry and soil fertility (Reich and Oleksyn, 2004). In this study, although soil N availability was thought to be the limitation to lucerne growth and thus there should be more interactions, yet lucerne BNF makes this effect on leaf stoichiometric ratios less obvious. The BNF may heavily affect the relationships between leaf and soil concerning N as lucerne is able to use symbiotically fixed N and is less dependent on soil mineral N (Yang et al., 2011a). In addition, soil C:N, N:P, and C:P hardly affected leaf C:N, C:P, and N:P in this study. This was different from Güsewell (2004), which indicated shoot N:P increased with N:P supply ratios. However, in some of soil layers, the correlation did exist. Further investigation on the link of stoichiometric ratios between plant and soil should be carried on.

## Conclusion

Leaf C:N, C:P, and N:P changed with stand age and cut of lucerne and they were generally the greatest in 8 year stand and in the second cut. In general, they were affected significantly and negatively by leaf TN and TP concentrations, but not by OC concentration. There were significant and positive correlations among leaf C:N, C:P, and N:P. However, leaf C:N, C:P, and N:P were hardly affected by soil features. Conclusively, lucerne C, N and P stoichiometry are age- and cut-specific, and regulated by leaf N, P, and stoichiometry, but there are few correlations with soil fertility. It should be helpful and practical for the persistence of lucerne production to apply optimal N fertilization to the stands of age 8 or older and shortly after the first cut of each year in this rainfed region.

## Conflict of Interest Statement

The authors declare that the research was conducted in the absence of any commercial or financial relationships that could be construed as a potential conflict of interest.
