# Supplementary material for: Stoichiometric Characteristics of Carbon, Nitrogen, and Phosphorus in Leaves of Differently Aged Lucerne (Medicago sativa) Stands
Source: Front Plant Sci. 2015 Dec 9;6:1062. doi: 10.3389/fpls.2015.01062 (PMC4673304; doi:10.3389/fpls.2015.01062)
Supplement: Supplementary file 1 [file Table_1.DOC]

**Stoichiometric characteristics of carbon, nitrogen and phosphorus in leaves of differently aged lucerne (*Medicago sativa*) stands**

Zhennan Wang, Jiaoyun Lu, Mei Yang, Huimin Yang*, Qingping Zhang

State Key Laboratory of Grassland Agro-ecosystems, College of Pastoral Agriculture Science and Technology, Lanzhou University, Lanzhou 730020, P. R. China

Zhennan Wang, [wangzn11@163.com](mailto:wangzn11@163.com)

Jiaoyun Lu, [lujy09@lzu.edu.cn](mailto:lujy09@lzu.edu.cn)

Mei Yang, [yangm14@lzu.edu.cn](http://mail.lzu.edu.cn/coremail/XT3/pab/view.jsp?sid=CAoFkyJJaorcrDNlidJJnYaxfloQDvVX&totalCount=19&view_no=11&puid=324&gid=4)

Huimin Yang, [huimyang@lzu.edu.cn](mailto:huimyang@lzu.edu.cn)

Qingping Zhang, [zhangqp2008@lzu.edu.cn](mailto:zhangqp2008@lzu.edu.cn)

***Correspondence:** Dr. Huimin Yang

Address: State Key Laboratory of Grassland Agro-ecosystems, College of Pastoral Agriculture Science and Technology, Lanzhou University, P. O. Box 61, 768 Jiayuguanxi Road, Lanzhou 730020, P. R. China

E-mail address: [huimyang@lzu.edu.cn](mailto:huimyang@lzu.edu.cn)

**Supplementary Information**

**FIGURE S1│Concentrations of OC (a), TN (b) and TP (c) in leaves of differently aged lucerne stands.** Different lowercase letters represent significant differences among ages at P<0.05. Different capital letters represent significant differences among cuts at P<0.05. The columns represent the arithmetic averages, and the bars represent the standard deviations.

**FIGURE S2│Correlations among concentrations of OC (a), TN (b) and TP (c) in the leaves of differently aged lucerne stands.** All data in four ages and three cuts were used for analysing the correlations. Only the case with significance is shown with a line in the figure.

**TABLE S1│Soil OC, TN and TP concentrations** **at different depths under differently aged lucerne stands.**

| **Soil depth (cm)** | **Cut** | **OC (g kg^-1^)** | | | | ***LSD*** | **TN (g kg^-1^)** | | | | ***LSD*** | **TP (g kg^-1^)** | | | | ***LSD*** |
| --- | --- | --- | --- | --- | --- | --- | --- | --- | --- | --- | --- | --- | --- | --- | --- | --- |
|  |  | **4** | **5** | **8** | **11** |  | **4** | **5** | **8** | **11** |  | **4** | **5** | **8** | **11** |  |
| 0-10 | 1^st^ | 8.13 | 10.97 | 8.81 | 7.50 | ***0.89*** | 1.14 | 1.37 | 1.41 | 1.27 | ***0.33*** | 0.78 | 0.86 | 0.83 | 0.78 | ***0.08*** |
|  | 2^nd^ | 6.43 | 8.44 | 8.00 | 8.60 | ***1.69*** | 0.95 | 1.20 | 1.31 | 1.19 | ***0.22*** | 0.73 | 0.80 | 0.89 | 0.80 | ***0.14*** |
|  | 3^rd^ | 7.14 | 9.08 | 7.71 | 7.86 | ***1.16*** | 1.30 | 1.14 | 1.01 | 0.67 | ***0.16*** | 0.91 | 0.82 | 0.92 | 0.81 | ***0.25*** |
|  | ***LSD*** | ***0.34*** | ***0.36*** | ***0.49*** | ***0.88*** | ***----*** | ***0.06*** | ***0.15*** | ***0.11*** | ***0.09*** | ***----*** | ***0.07*** | ***0.10*** | ***0.05*** | ***0.08*** | ***----*** |
| 10-20 | 1^st^ | 7.45 | 9.07 | 6.93 | 6.74 | ***0.99*** | 1.10 | 1.10 | 1.05 | 1.12 | ***0.25*** | 0.72 | 0.85 | 0.77 | 0.63 | ***0.11*** |
|  | 2^nd^ | 6.07 | 6.94 | 6.42 | 6.23 | ***1.53*** | 0.87 | 0.95 | 1.11 | 1.10 | ***0.23*** | 0.75 | 0.78 | 0.88 | 0.64 | ***0.15*** |
|  | 3^rd^ | 6.73 | 5.95 | 6.10 | 4.98 | ***1.13*** | 1.19 | 0.71 | 0.95 | 0.63 | ***0.22*** | 0.85 | 0.98 | 0.89 | 0.82 | ***0.18*** |
|  | ***LSD*** | ***0.41*** | ***0.50*** | ***0.63*** | ***0.59*** | ***----*** | ***0.11*** | ***0.08*** | ***0.10*** | ***0.11*** | ***----*** | ***0.08*** | ***0.03*** | ***0.050*** | ***0.09*** | ***----*** |
| 20-30 | 1^st^ | 5.76 | 6.36 | 5.62 | 5.40 | ***0.68*** | 0.99 | 0.95 | 0.93 | 0.91 | ***0.20*** | 0.69 | 0.72 | 0.71 | 0.64 | ***0.12*** |
|  | 2^nd^ | 5.36 | 5.61 | 5.64 | 5.89 | ***0.62*** | 0.89 | 0.96 | 0.80 | 0.98 | ***0.28*** | 0.72 | 0.96 | 0.87 | 0.93 | ***0.15*** |
|  | 3^rd^ | 5.29 | 5.12 | 4.85 | 4.61 | ***0.79*** | 1.05 | 0.76 | 0.50 | 0.65 | ***0.22*** | 0.96 | 0.82 | 0.91 | 0.76 | ***0.19*** |
|  | ***LSD*** | ***0.39*** | ***0.16*** | ***0.35*** | ***0.27*** | ***----*** | ***0.11*** | ***0.12*** | ***0.08*** | ***0.09*** | ***----*** | ***0.07*** | ***0.08*** | ***0.05*** | ***0.07*** | ***----*** |
| 30-60 | 1^st^ | 4.85 | 6.21 | 6.51 | 5.46 | ***2.13*** | 1.06 | 0.74 | 0.87 | 0.92 | ***0.26*** | 0.66 | 0.73 | 0.70 | 0.65 | ***0.09*** |
|  | 2^nd^ | 5.64 | 6.12 | 6.44 | 6.93 | ***1.30*** | 0.66 | 0.84 | 0.87 | 0.83 | ***0.15*** | 0.65 | 0.99 | 0.76 | 0.67 | ***0.15*** |
|  | 3^rd^ | 4.59 | 4.84 | 6.09 | 6.10 | ***0.89*** | 1.00 | 0.73 | 0.82 | 0.61 | ***0.14*** | 0.80 | 0.82 | 0.77 | 0.78 | ***0.19*** |
|  | ***LSD*** | ***1.06*** | ***0.24*** | ***0.31*** | ***0.70*** | ***----*** | ***0.12*** | ***0.07*** | ***0.04*** | ***0.08*** | ***----*** | ***0.05*** | ***0.09*** | ***0.05*** | ***0.06*** | ***----*** |
| 60-90 | 1^st^ | 6.75 | 4.79 | 6.57 | 5.74 | ***1.07*** | 1.25 | 0.84 | 1.15 | 1.06 | ***0.25*** | 0.74 | 0.75 | 0.71 | 0.69 | ***0.11*** |
|  | 2^nd^ | 5.79 | 4.38 | 5.28 | 5.24 | ***0.74*** | 0.63 | 0.88 | 0.72 | 0.67 | ***0.36*** | 0.80 | 0.90 | 0.77 | 0.64 | ***0.16*** |
|  | 3^rd^ | 5.62 | 4.28 | 5.18 | 6.38 | ***1.19*** | 1.05 | 0.55 | 0.72 | 0.50 | ***0.16*** | 0.85 | 0.93 | 0.82 | 0.82 | ***0.21*** |
|  | ***LSD*** | ***0.53*** | ***0.47*** | ***0.15*** | ***0.51*** | ***----*** | ***0.10*** | ***0.09*** | ***0.11*** | ***0.15*** | ***----*** | ***0.10*** | ***0.04*** | ***0.07*** | ***0.07*** | ***----*** |
| ***LSD_depth_*** | 1^st^ | ***1.94*** | ***1.02*** | ***0.39*** | ***0.99*** | ***----*** | ***0.25*** | ***0.31*** | ***0.22*** | ***0.21*** | ***----*** | ***0.08*** | ***0.09*** | ***0.09*** | ***0.13*** | ***----*** |
|  | 2^nd^ | ***0.94*** | ***0.70*** | ***1.21*** | ***1.74*** | ***----*** | ***0.23*** | ***0.17*** | ***0.22*** | ***0.33*** | ***----*** | ***0.16*** | ***0.16*** | ***0.12*** | ***0.13*** | ***----*** |
|  | 3^rd^ | ***0.89*** | ***0.71*** | ***1.00*** | ***1.33*** | ***----*** | ***0.20*** | ***0.21*** | ***0.16*** | ***0.13*** | ***----*** | ***0.23*** | ***0.21*** | ***0.13*** | ***0.21*** | ***----*** |
| Age | |  | *P=0.009* | |  |  |  | *P<0.001* | |  |  |  | *P<0.001* | |  |  |
| Cut | |  | *P<0.001* | |  |  |  | *P<0.001* | |  |  |  | *P<0.001* | |  |  |
| Depth | |  | *P<0.001* | |  |  |  | *P<0.001* | |  |  |  | *P=0.003* | |  |  |
| Age×Cut | |  | *P<0.001* | |  |  |  | *P<0.001* | |  |  |  | *P=0.042* | |  |  |
| Age×Depth | |  | *P<0.001* | |  |  |  | *P<0.001* | |  |  |  | *P=0.088* | |  |  |
| Cut×Depth | |  | *P<0.001* | |  |  |  | *P=0.001* | |  |  |  | *P=0.006* | |  |  |
| Age×Cut×Depth | |  | *P=0.031* | |  |  |  | *P=0.022* | |  |  |  | *P=0.025* | |  |  |

*4, 5, 8 and 11 refer to stand age in year and 1^st^, 2^nd^ and 3^rd^ refer to the cut of one age.*

*LSD was at the 0.05 level.*

**TABLE S2│Soil NH_4_^+^, NO_3_^-^ and AP concentrations at different depths under differently aged lucerne stands.**

| **Soil depth (cm)** | **Cut** | **NH_4_^+^ (mg kg^-1^)** | | | | ***LSD*** | **NO_3_^-^ (mg kg^-1^)** | | | | ***LSD*** | **AP (mg kg^-1^)** | | | | ***LSD*** |
| --- | --- | --- | --- | --- | --- | --- | --- | --- | --- | --- | --- | --- | --- | --- | --- | --- |
|  |  | **4** | **5** | **8** | **11** |  | **4** | **5** | **8** | **11** |  | **4** | **5** | **8** | **11** |  |
| 0-10 | 1^st^ | 54.0 | 53.2 | 47.1 | 39.9 | ***5.8*** | 1.22 | 1.84 | 2.03 | 6.13 | ***2.64*** | 77.1 | 41.1 | 53.7 | 47.4 | ***30.6*** |
|  | 2^nd^ | 29.9 | 37.8 | 40.1 | 30.2 | ***1.6*** | 10.50 | 12.85 | 6.96 | 8.28 | ***8.45*** | 23.2 | 59.6 | 41.4 | 16.2 | ***13.7*** |
|  | 3^rd^ | 30.9 | 35.9 | 31.0 | 37.3 | ***1.9*** | 3.20 | 2.06 | 2.90 | 3.67 | ***4.71*** | 23.6 | 41.1 | 14.1 | 54.7 | ***28.1*** |
|  | ***LSD*** | ***1.5*** | ***6.6*** | ***1.6*** | ***3.3*** | ***----*** | ***2.63*** | ***7.01*** | ***4.33*** | ***8.74*** | ***----*** | ***18.7*** | ***12.9*** | ***17.3*** | ***45.4*** | ***----*** |
| 10-20 | 1^st^ | 49.7 | 48.6 | 43.9 | 35.3 | ***4.4*** | 1.58 | 1.31 | 0.69 | 3.63 | ***1.17*** | 66.6 | 26.0 | 77.1 | 25.0 | ***26.6*** |
|  | 2^nd^ | 31.6 | 39.0 | 41.4 | 29.5 | ***2.2*** | 10.62 | 8.79 | 4.44 | 5.90 | ***3.85*** | 25.0 | 39.7 | 37.9 | 14.8 | ***19.4*** |
|  | 3^rd^ | 32.0 | 34.3 | 31.9 | 36.8 | ***1.6*** | 6.16 | 0.76 | 2.94 | 0.71 | ***1.41*** | 25.0 | 31.6 | 16.6 | 14.5 | ***10.5*** |
|  | ***LSD*** | ***3.0*** | ***5.0*** | ***2.0*** | ***1.2*** | ***----*** | ***2.44*** | ***2.32*** | ***2.75*** | ***2.90*** | ***----*** | ***30.1*** | ***13.5*** | ***22.9*** | ***13.5*** | ***----*** |
| 20-30 | 1^st^ | 46.7 | 44.5 | 40.3 | 32.5 | ***2.4*** | 1.44 | 0.11 | 0.61 | 5.65 | ***3.46*** | 42.8 | 22.9 | 23.2 | 16.6 | ***12.0*** |
|  | 2^nd^ | 30.8 | 38.9 | 42.8 | 30.7 | ***1.4*** | 7.28 | 5.29 | 1.62 | 4.58 | ***5.13*** | 19.4 | 27.1 | 41.8 | 6.4 | ***20.5*** |
|  | 3^rd^ | 32.3 | 35.6 | 32.2 | 37.3 | ***0.9*** | 5.60 | 0.10 | 3.47 | 0.90 | ***2.47*** | 19.4 | 31.3 | 13.8 | 30.2 | ***18.6*** |
|  | ***LSD*** | ***0.9*** | ***1.7*** | ***0.9*** | ***2.9*** | ***----*** | ***2.99*** | ***6.10*** | ***1.51*** | ***4.27*** | ***----*** | ***18.5*** | ***8.7*** | ***25.2*** | ***17.8*** | ***----*** |
| 30-60 | 1^st^ | 42.3 | 46.1 | 38.3 | 30.6 | ***5.0*** | 0.11 | 0.43 | 1.35 | 1.46 | ***0.44*** | 31.3 | 17.3 | 22.2 | 18.0 | ***6.09*** |
|  | 2^nd^ | 29.2 | 37.8 | 40.4 | 30.8 | ***1.4*** | 4.54 | 3.50 | 3.47 | 4.06 | ***2.56*** | 20.8 | 27.1 | 46.3 | 12.0 | ***17.8*** |
|  | 3^rd^ | 31.5 | 35.1 | 30.6 | 37.3 | ***1.0*** | 2.13 | 0.13 | 1.69 | 1.22 | ***1.19*** | 10.6 | 28.5 | 11.3 | 28.1 | ***12.1*** |
|  | ***LSD*** | ***1.2*** | ***5.7*** | ***1.4*** | ***2.5*** | ***----*** | ***1.93*** | ***2.40*** | ***0.82*** | ***1.44*** | ***----*** | ***7.3*** | ***13.5*** | ***17.1*** | ***15.0*** | ***----*** |
| 60-90 | 1^st^ | 41.4 | 42.5 | 38.9 | 28.3 | ***1.7*** | 1.49 | 0.06 | 1.10 | 2.92 | ***1.56*** | 20.4 | 13.8 | 19.7 | 24.3 | ***5.7*** |
|  | 2^nd^ | 30.2 | 37.9 | 39.9 | 31.5 | ***1.5*** | 3.73 | 0.77 | 0.88 | 4.16 | ***4.17*** | 18.0 | 19.7 | 41.1 | 9.6 | ***19.5*** |
|  | 3^rd^ | 31.1 | 34.6 | 30.6 | 36.5 | ***2.1*** | 2.07 | 0.73 | 1.49 | 1.62 | ***1.88*** | 17.3 | 19.7 | 6.9 | 25.3 | ***12.9*** |
|  | ***LSD*** | ***2.0*** | ***2.5*** | ***1.2*** | ***1.4*** | ***----*** | ***2.51*** | ***0.62*** | ***1.14*** | ***5.20*** | ***----*** | ***12.4*** | ***7.3*** | ***23.4*** | ***10.9*** | ***----*** |
| ***LSD_depth_*** | ***1^st^*** | ***1.5*** | ***6.9*** | ***1.6*** | ***3.4*** | ***----*** | ***0.19*** | ***1.81*** | ***0.88*** | ***3.61*** | ***----*** | ***25.0*** | ***8.5*** | ***13.4*** | ***22.8*** | ***----*** |
|  | ***2^nd^*** | ***2.2*** | ***1.4*** | ***1.4*** | ***1.2*** | ***----*** | ***2.74*** | ***6.43*** | ***3.29*** | ***6.50*** | ***----*** | ***9.4*** | ***10.6*** | ***30.0*** | ***12.3*** | ***----*** |
|  | ***3^rd^*** | ***1.3*** | ***2.3*** | ***1.0*** | ***1.1*** | ***----*** | ***2.88*** | ***2.02*** | ***1.87*** | ***3.22*** | ***----*** | ***13.6*** | ***12.0*** | ***8.0*** | ***27.8*** | ***----*** |
| Age | |  | *P<0.001* | |  |  |  | *P<0.001* | |  |  |  | *P<0.001* | |  |  |
| Cut | |  | *P<0.001* | |  |  |  | *P<0.001* | |  |  |  | *P<0.001* | |  |  |
| Depth | |  | *P<0.001* | |  |  |  | *P<0.001* | |  |  |  | *P<0.001* | |  |  |
| Age×Cut | |  | *P<0.001* | |  |  |  | *P<0.001* | |  |  |  | *P<0.001* | |  |  |
| Age×Depth | |  | *P=0.265* | |  |  |  | *P=0.048* | |  |  |  | *P=0.003* | |  |  |
| Cut×Depth | |  | *P<0.001* | |  |  |  | *P<0.001* | |  |  |  | *P<0.001* | |  |  |
| Age×Cut×Depth | |  | *P=0.443* | |  |  |  | *P=0.335* | |  |  |  | *P=0.012* | |  |  |

*4, 5, 8 and 11 refer to stand age in year and 1^st^, 2^nd^ and 3^rd^ refer to the cut of one age.*

*LSD was at the 0.05 level.*

***TABLE S3│Soil C:N, C:P and N:P at different depths under differently aged lucerne stands.***

| **Soil depth (cm)** | **Cut** | **C:N** | | | | ***LSD*** | **C:P** | | | | ***LSD*** | **N:P** | | | | ***LSD*** |
| --- | --- | --- | --- | --- | --- | --- | --- | --- | --- | --- | --- | --- | --- | --- | --- | --- |
|  |  | **4** | **5** | **8** | **11** |  | **4** | **5** | **8** | **11** |  | **4** | **5** | **8** | **11** |  |
| 0-10 | 1^st^ | 7.21 | 8.19 | 6.28 | 5.95 | ***1.71*** | 10.40 | 12.90 | 10.64 | 9.60 | *1.88* | 1.45 | 1.62 | 1.70 | 1.62 | *0.45* |
|  | 2^nd^ | 6.83 | 7.08 | 6.12 | 7.16 | ***1.25*** | 8.88 | 10.57 | 8.96 | 10.81 | *2.55* | 1.30 | 1.51 | 1.47 | 1.50 | *0.33* |
|  | 3^rd^ | 5.51 | 8.00 | 7.66 | 11.72 | ***1.13*** | 7.98 | 11.36 | 8.46 | 9.79 | *3.00* | 1.45 | 1.43 | 1.10 | 0.84 | *0.35* |
|  | ***LSD*** | ***1.59*** | ***1.85*** | ***0.75*** | ***1.47*** | ***----*** | ***2.17*** | ***3.39*** | ***1.19*** | ***3.31*** | ***----*** | ***0.24*** | ***0.66*** | ***0.25*** | ***0.33*** | ***----*** |
| 10-20 | 1^st^ | 6.82 | 8.24 | 6.69 | 6.05 | ***1.07*** | 10.35 | 10.63 | 8.96 | 10.81 | *1.38* | 1.52 | 1.29 | 1.36 | 1.79 | *0.30* |
|  | 2^nd^ | 7.01 | 7.33 | 5.92 | 5.81 | ***2.32*** | 8.21 | 8.97 | 7.41 | 9.87 | *2.84* | 1.17 | 1.22 | 1.26 | 1.73 | *0.30* |
|  | 3^rd^ | 5.75 | 8.44 | 6.43 | 7.88 | ***1.46*** | 8.11 | 6.06 | 6.87 | 6.13 | *1.95* | 1.41 | 0.72 | 1.07 | 0.78 | *0.23* |
|  | ***LSD*** | ***1.74*** | ***1.27*** | ***2.32*** | ***1.72*** | ***----*** | ***2.69*** | ***1.06*** | ***2.40*** | ***2.57*** | ***----*** | ***0.22*** | ***0.14*** | ***0.27*** | ***0.45*** | ***----*** |
| 20-30 | 1^st^ | 5.88 | 6.68 | 6.08 | 5.97 | ***1.19*** | 8.35 | 8.84 | 8.04 | 8.48 | *1.49* | 1.44 | 1.32 | 1.33 | 1.43 | *0.36* |
|  | 2^nd^ | 6.14 | 5.91 | 7.05 | 6.09 | ***1.35*** | 7.44 | 5.90 | 6.49 | 6.33 | *1.15* | 1.23 | 1.01 | 0.93 | 1.05 | *0.37* |
|  | 3^rd^ | 5.04 | 7.02 | 9.97 | 7.16 | ***3.28*** | 5.53 | 6.33 | 5.35 | 6.15 | *1.54* | 1.11 | 0.94 | 0.55 | 0.86 | *0.30* |
|  | ***LSD*** | ***1.54*** | ***2.15*** | ***3.41*** | ***1.54*** | ***----*** | ***1.43*** | ***1.44*** | ***1.85*** | ***1.15*** | ***----*** | ***0.47*** | ***0.35*** | ***0.34*** | ***0.27*** | ***----*** |
| 30-60 | 1^st^ | 4.54 | 8.63 | 7.47 | 5.89 | ***1.97*** | 7.19 | 8.49 | 9.29 | 8.34 | *2.56* | 1.59 | 1.00 | 1.24 | 1.41 | *0.28* |
|  | 2^nd^ | 8.62 | 7.28 | 7.43 | 8.39 | ***2.08*** | 8.71 | 6.22 | 8.47 | 10.29 | *1.85* | 1.03 | 0.86 | 1.15 | 1.23 | *0.29* |
|  | 3^rd^ | 4.60 | 6.70 | 7.41 | 10.09 | ***1.20*** | 5.73 | 6.00 | 7.97 | 7.93 | *1.62* | 1.25 | 0.91 | 1.08 | 0.79 | *0.33* |
|  | ***LSD*** | ***2.56*** | ***2.15*** | ***1.56*** | ***0.89*** | ***----*** | ***3.26*** | ***1.30*** | ***1.59*** | ***2.01*** | ***----*** | ***0.40*** | ***0.37*** | ***0.21*** | ***0.25*** | ***----*** |
| 60-90 | 1^st^ | 5.36 | 5.80 | 5.80 | 5.49 | ***1.23*** | 9.18 | 6.38 | 9.30 | 8.37 | *1.94* | 1.71 | 1.11 | 1.61 | 1.56 | *0.43* |
|  | 2^nd^ | 9.83 | 5.05 | 7.57 | 8.58 | ***4.55*** | 7.38 | 4.90 | 6.86 | 8.22 | *1.85* | 0.83 | 0.98 | 0.93 | 1.04 | *0.54* |
|  | 3^rd^ | 5.34 | 8.05 | 7.22 | 13.12 | ***4.26*** | 6.86 | 4.59 | 6.32 | 7.78 | *2.12* | 1.29 | 0.59 | 0.88 | 0.61 | *0.38* |
|  | ***LSD*** | ***3.95*** | ***2.97*** | ***2.63*** | ***5.41*** | ***----*** | ***2.98*** | ***1.35*** | ***1.62*** | ***2.05*** | ***----*** | ***0.61*** | ***0.23*** | ***0.22*** | ***0.67*** | ***----*** |
| ***LSD_depth_*** | ***1^st^*** | ***1.57*** | ***1.81*** | ***1.11*** | ***1.07*** | ***----*** | ***2.46*** | ***1.39*** | ***1.54*** | ***1.75*** | ***----*** | ***0.32*** | ***0.38*** | ***0.29*** | ***0.41*** | ***----*** |
|  | ***2^nd^*** | ***3.35*** | ***1.49*** | ***2.39*** | ***2.48*** | ***----*** | ***2.31*** | ***1.25*** | ***1.96*** | ***2.50*** | ***----*** | ***0.43*** | ***0.28*** | ***0.23*** | ***0.47*** | ***----*** |
|  | ***3^rd^*** | ***1.15*** | ***2.45*** | ***2.54*** | ***3.38*** | ***----*** | ***2.30*** | ***2.36*** | ***1.27*** | ***2.05*** | ***----*** | ***0.37*** | ***0.39*** | ***0.18*** | ***0.24*** | ***----*** |
| Age | |  | *P<0.001* | |  |  |  | *P=0.009* | |  |  |  | *P<0.001* | |  |  |
| Cut | |  | *P<0.001* | |  |  |  | *P<0.001* | |  |  |  | *P<0.001* | |  |  |
| Depth | |  | *P=0.047* | |  |  |  | *P<0.001* | |  |  |  | *P<0.001* | |  |  |
| Age×Cut | |  | *P<0.001* | |  |  |  | *P=0.017* | |  |  |  | *P<0.001* | |  |  |
| Age×Depth | |  | *P<0.001* | |  |  |  | *P<0.001* | |  |  |  | *P<0.001* | |  |  |
| Cut×Depth | |  | *P<0.001* | |  |  |  | *P=0.006* | |  |  |  | *P=0.002* | |  |  |
| Age×Cut×Depth | |  | *P=0.004* | |  |  |  | *P=0.092* | |  |  |  | *P=0.043* | |  |  |

*4, 5, 8 and 11 refer to stand age in year and 1^st^, 2^nd^ and 3^rd^ refer to the cut of one age.*

*LSD was at the 0.05 level.*
